# Effect of Surgical Position and Preoperative Antihypertensive Medication on the Incidence of Intraoperative Hypotension in Patients Undergoing Spine Surgery

**DOI:** 10.5812/aapm-161684

**Published:** 2025-05-21

**Authors:** Yei Heum Park, Jae Hong Park, Daeyun Choi, Min Woo Lee, Daeseok Oh, Sung Ho Moon, Ji Yeon Kwon, Myungjin Ko

**Affiliations:** 1 Department of Anesthesiology and Pain Medicine, Inje University Haeundae Paik Hospital, Busan, Korea; 2 Department of Anesthesiology and Pain Medicine, Asan Medical Center, University of Ulsan College of Medicine, Seoul, Korea

**Keywords:** Antihypertensive Agents, General Anesthesia, Hypotension, Prone Position, Risk Factors, Spine Surgery

## Abstract

**Background:**

Antihypertensive medications taken before surgery are associated with increased intraoperative hypotension, and patient positioning can further influence hemodynamics during surgery. However, the combined effects of antihypertensive medication use and patient positioning on intraoperative hypotension during spine surgery have not been clearly established.

**Objectives:**

This study aimed to investigate the incidence of hypotension in patients undergoing spine surgery according to surgical position, antihypertensive drug use, and patient characteristics through a retrospective analysis of medical records.

**Methods:**

This retrospective study analyzed 4,973 patients who had undergone spine surgery. Demographic data, medical history, antihypertensive medication use before surgery, and anesthetic information, including blood pressure during surgery, were collected from electronic medical records (EMRs). The incidence of hypotension according to surgical positioning (supine vs. prone) and antihypertensive medication use was investigated.

**Results:**

The incidence of intraoperative hypotension was higher in patients positioned prone (supine: 19.06% vs. prone: 24.91%) and among those taking more antihypertensive medications (no medication: 19.49%; one medication: 25.18%; two or more medications: 32.97%). Logistic regression indicated that patients with a history of hypertension undergoing surgery in the prone position had a significantly greater risk of hypotension [odds ratio (OR) = 1.407] and severe hypotension (OR = 1.940) compared with those with no history of hypertension undergoing surgery in the supine position. Older age, longer anesthesia duration, cervical surgical site, and the use of multiple antihypertensive agents were associated with an increased risk of intraoperative hypotension. In particular, taking two or more antihypertensive drugs (OR = 1.601) and undergoing surgery in the prone position (OR = 1.505) were independent predictors of hypotension and severe hypotension during spine surgery.

**Conclusions:**

Preoperative use of two or more antihypertensive medications increases the risk of intraoperative hypotension, and spine surgery in the prone position increases the risk of severe hypotension.

## 1. Background

Spinal surgery is typically performed to address degenerative conditions such as spinal stenosis and herniated intervertebral discs, which are more prevalent among the elderly population (1). Patients undergoing these procedures often present with multiple chronic illnesses, with hypertension being particularly common (2, 3). Hypertension can influence a patient's hemodynamic state during the perioperative period, and several studies have indicated that the administration of antihypertensive medications prior to surgery may impact the incidence of intraoperative hypotension (4-6). Notably, angiotensin-converting enzyme (ACE) inhibitors and angiotensin receptor blockers (ARBs) markedly increase the frequency of hypotension during surgical procedures (7).

Spinal surgery may be conducted in various positions, including supine, lateral, and prone, depending on the specific surgical site and technique employed (8). The prone position is the most frequently utilized for spine surgeries, and it can have significant effects on the patient's hemodynamic state. Transitioning from a supine to a prone position following the induction of anesthesia can lead to unexpected complications, with hemodynamic alterations being a critical concern for patient safety (9-11). Backofen reported a substantial decrease in Cardiac Index and stroke volume when patients were repositioned to the prone position, which was associated with a significant increase in systemic and pulmonary vascular resistance (12).

Hypotension during surgery is a significant complication that can lead to severe and potentially fatal outcomes. Therefore, it is crucial to understand the factors that may contribute to its occurrence during surgical procedures (13). Despite its importance, limited research examining the impact of patient positioning — specifically, supine versus prone — on the incidence of intraoperative hypotension among individuals taking antihypertensive medication prior to surgery has been conducted.

## 2. Objectives

This study aimed to investigate the incidence of hypotension in patients undergoing spine surgery according to surgical position, the use of antihypertensive drugs, and various patient characteristics through a retrospective analysis of medical records.

## 3. Methods

### 3.1. Study Population

This retrospective study analyzed the electronic medical records (EMRs) of patients who underwent elective spine surgery at Haeundae Paik Hospital between March 2010 and July 2021. The surgical procedures included arthrodesis, open diskectomy, and laminectomy, while minimally invasive surgeries using endoscopic techniques and simple procedures such as nerve blocks were excluded. In total, 5,058 patients were initially considered for inclusion; however, 85 were excluded due to incomplete or inaccurate data, resulting in a final cohort of 4,973 patients. Although this is a retrospective study that did not involve a predetermined sample size calculation, a post hoc power analysis confirmed that the sample size was adequate to detect significant differences in the incidence of hypotension across the study groups. All patients underwent spine surgery under general anesthesia, with the variables analyzed including surgical position and preoperative antihypertensive medication.

### 3.2. Study Design

This study was conducted as a retrospective observational analysis. All patient data were retrieved from the hospital’s EMR system. This study was registered with the Clinical Research Information Service (CRIS number: KCT0009892) and received approval from the Institutional Review Board (IRB) of Haeundae Paik Hospital (IRB number: HPIRB 2022-05-048). The requirement for written informed consent was waived by the IRB due to the retrospective nature of this study.

### 3.3. Variables and Measurements

Demographic data of the patients, including age, sex, weight, height, American Society of Anesthesiology physical status (ASA-PS) classification, and history of hypertension, cardiac disease, and diabetes mellitus (DM), were collected. The type and number of antihypertensive medications that patients were currently taking were also examined.

Blood pressure was monitored either continuously via an arterial line or at 3-min intervals using non-invasive blood pressure methods, and recorded at 5-min intervals in the anesthesia recording chart. The baseline MAP was defined as the initial blood pressure measured upon entry to the operating room. In accordance with institutional clinical practice, intraoperative blood pressure was managed with the goal of maintaining values within ± 20% of this baseline through titration of fluid administration, inhalational anesthetics, and remifentanil infusion. In this study, intraoperative hypotension was defined as any MAP < 60 mmHg, and severe hypotension as MAP < 50 mmHg that persisted for at least 5 min.

Anesthesia was induced using propofol at a dosage of 1 to 2 mg/kg. Although this was a retrospective study and individual titration details were not consistently available, propofol dosing was generally adjusted at the discretion of the attending anesthesiologist with consideration of each patient’s condition. At our institution, it is common practice to administer 1.0 - 1.5 mg/kg to patients with ASA-PS III or above or those aged 65 years and older, and 1.5 - 2.0 mg/kg to younger or healthier individuals. This reflects efforts to minimize hemodynamic instability during induction, given the known hypotensive effects of propofol. To maintain anesthesia, either sevoflurane or desflurane was administered at 0.8 to 1.2 minimum alveolar concentration, alongside remifentanil at a dosage of 0.03 to 0.1 mcg/kg/min. The Bispectral Index was maintained within 40 - 60 to ensure adequate depth of anesthesia.

Patients were categorized into groups based on their surgical positions and the presence or absence of a history of hypertension: Group 1 (supine position without a history of hypertension), group 2 (supine position with a history of hypertension), group 3 (prone position without a history of hypertension), and group 4 (prone position with a history of hypertension).

Further analysis was conducted according to the type and number of antihypertensive medications taken. Antihypertensive medications were classified into five categories: Angiotensin-converting enzyme inhibitors, ARBs, calcium channel blockers (CCBs), beta-blockers (BBs), and diuretics. The number of antihypertensive medications taken prior to surgery was also examined and categorized as no medication, one medication, or two or more medications.

### 3.4. Statistical Analysis

Statistical analyses were conducted using SPSS version 25.0 (IBM Corp., Armonk, NY). Logistic regression was employed to assess the factors associated with intraoperative hypotension. Univariable analysis was initially performed for individual variables, followed by multivariable analysis utilizing backward elimination for variables with P-values < 0.05. Odds ratios (ORs) with 95% confidence intervals (CIs) were calculated to evaluate the strength of association between risk factors and hypotension occurrence.

The incidences of hypotension (MAP < 60 mmHg) and severe hypotension (MAP < 50 mmHg) were analyzed. Risk factors, including age, sex, weight, height, antihypertensive medication usage, and surgical position, were examined for their impact on hypotension during surgery. A P-value < 0.05 was deemed statistically significant.

## 4. Results

**Figure 1.**
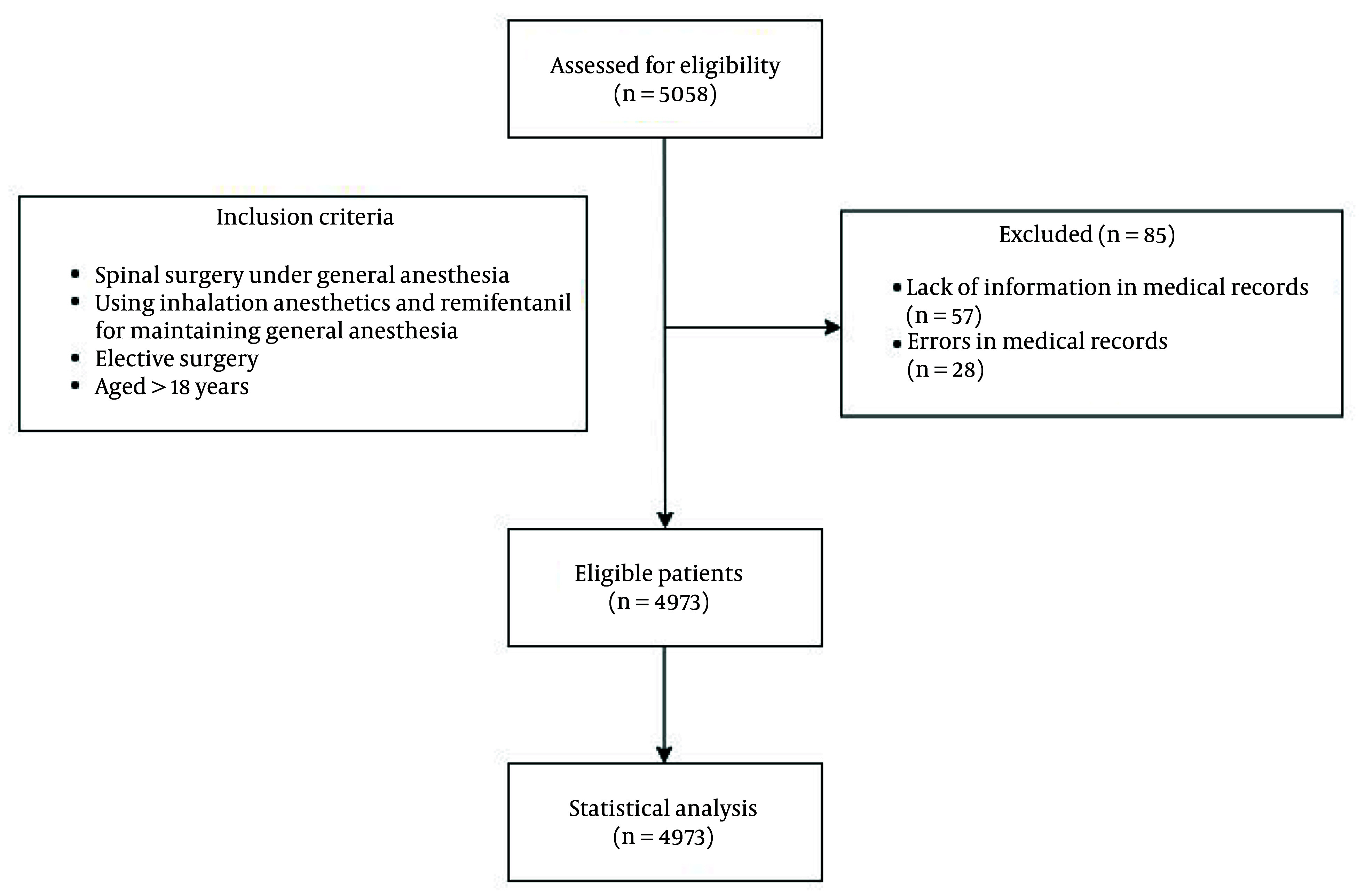
Flowchart of the study

In total, 5,058 patients who underwent spinal surgery under general anesthesia were included in this study. Due to insufficient or inaccurate information in their medical records, 85 were excluded, resulting in 4,973 patients included in the final analysis (Figure 1). The surgical positions of the patients were categorized as supine (25.8%) and prone (74.2%), while the operation sites included the cervical (26.68%), thoracic (7.06%), and lumbar (66.26%) regions. Among all patients, 1,697 (34.1%) had a history of hypertension. Furthermore, 3,048 patients (61.3%) were not taking antihypertensive medication prior to surgery, 830 (16.7%) were taking only one type, and 1,095 (22.0%) were taking two or more types (Table 1). 

**Table 1.** Patients Characteristics (N = 4973)

Characteristics	Mean ± SD or No. (%)
**Duration of anesthesia (min)**	200.41 ± 97.25
**Age (y)**	58.70 ± 14.33
**Height (cm)**	162.76 ± 9.64
**Site of operation**	
Cervical	1327 (26.68)
Thoracic	351 (7.06)
Lumbar	3295 (66.26)
**Amount of bleeding (mL)**	357.09 ± 548.07
**Hypotension during operation ** ^**a**^	
No	3809 (76.6)
Yes	1164 (23.4)
**Hypertension**	
No	3276 (65.9)
Yes	1697 (34.1)
**ASA-PS**	
1	1077 (21.7)
2	2667 (53.7)
3	1148 (23.1)
4	80 (1.6)
5	1 (0.0)
**ACE inhibitor**	
No	4850 (97.5)
Yes	12 3(2.5)
**BB**	
No	4415 (88.8)
Yes	558 (11.2)
**Diuretics**	
No	4367 (87.8)
Yes	606 (12.2)
**Duration of operation (min)**	143.99 ± 87.63
**Weight (kg)**	65.18 ± 12.41
**BMI**	24.52 ± 3.62
**Surgical position**	
Supine	1284 (25.8)
Prone	3689 (74.2)
**Sex**	
Male	2241 (45.1)
Female	2732 (54.9)
**Cardiac disease**	
No	4587 (92.2)
Yes	386 (7.8)
**DM**	
No	4105 (82.5)
Yes	868 (17.5)
**Antihypertensive drug count**	
0	3048 (61.3)
1	830 (16.7)
2 or more	1095 (22.0)
**ARB**	
No	3776 (75.9)
Yes	1197 (24.1)
**CCB**	
No	3969 (79.8)
Yes	1004 (20.2)

Abbreviations: ASA-PS, American Society of Anesthesiology physical status; ACE, angiotensin-converting enzyme; BB, beta blocker; BMI, Body Mass Index; DM, diabetes mellitus; ARB, angiotensin receptor blocker; CCB, calcium channel blocker.

^a^ Hypotension is defined as a mean arterial pressure < 60 mmHg.

The overall incidence of hypotension was 23.41%, with severe hypotension occurring in 4.4% of cases. When examining intraoperative hypotension in relation to antihypertensive medication count and surgical position, the incidence was significantly higher in patients positioned prone compared with those positioned supine (supine: 19.06% vs. prone: 24.91%; P < 0.001). In addition, intraoperative hypotension significantly increased with the number of antihypertensive drugs taken before surgery (no medications: 19.49%; one medication: 25.18%; two or more medications: 32.97%; P < 0.001; (Table 2). Table 3 presents the occurrence of hypotension during surgery based on the type of antihypertensive drug. Patients taking ARBs, CCBs, BBs, and diuretics had a significantly higher incidence of hypotension compared with those not taking them (P < 0.001 for all). Although ACE inhibitors are commonly associated with intraoperative hypotension, our findings did not show a significant increase in hypotension frequency with their use (7). Although individual drug classes showed varying associations with hypotension, direct comparisons between classes (e.g., ARBs vs. BBs) were not statistically tested in this study. Group 4 had the highest incidence of both hypotension (30.12%) and severe hypotension (6.29%;) (Table 4). 

**Table 2.** Incidence of Hypotension and Severe Hypotension During Surgery ^a, ^^b^

Categories	Hypotension	Severe Hypotension	P-Value ^c^ (Hypotension)	P-Value ^c^ (Severe Hypotension)
**Overall (N = 4973)**	1164 (23.41)	219 (4.40)	-	-
**Antihypertensive medication count**			< 0.001	< 0.001
0 (n = 3048)	594 (19.49)	102 (3.35)		
1 (n = 830)	209 (25.18)	34 (4.10)		
2 or more (n = 1095)	361 (32.97)	83 (7.58)		
**Surgical position**			< 0.001	< 0.001
Supine (n = 1280)	244 (19.06)	34 (2.65)		
Prone (n = 3693)	920 (24.91)	185 (5.01)		

^a^ Hypotension is defined as mean arterial pressure < 60 mmHg, and severe hypotension is defined as mean arterial pressure < 50 mmHg.

^b^ Values are expressed as No. (%).

^c^ P-values were calculated using the chi-squared test.

**Table 3.** Incidence of Intraoperative Hypotension According to Antihypertensive Drug Type ^a, ^^b^

Variables	Total	No Hypotension	Hypotension	P-Value ^c^
**ACE inhibitor**				0.120
No	4850 (97.5)	3722 (76.7)	1128 (23.3)	
Yes	123 (2.5)	87 (70.7)	36 (29.3)	
**ARB**				< 0.001
No	3776 (75.9)	2983 (79.0)	793 (21.0)	
Yes	1197 (24.1)	826 (69.0)	371 (31.0)	
**CCB**				< 0.001
No	3969 (79.8)	3100 (78.1)	869 (21.9)	
Yes	1004 (20.2)	709 (70.6)	295 (29.4)	
**BB**				< 0.001
No	4415 (88.8)	3433 (77.85)	982 (22.2)	
Yes	558 (11.2)	376 (67.4)	188 (32.6)	
**Diuretics**				< 0.001
No	4367 (87.8)	3414 (78.2)	953 (21.8)	
Yes	606 (12.2)	395 (65.2)	211 (34.8)	

Abbreviations: ACE, angiotensin-converting enzyme; ARB, angiotensin receptor blocker; CCB, calcium channel blocker; BB, beta blocker.

^a^ Hypotension is defined as mean arterial pressure < 60 mmHg.

^b^ Values are expressed as the N. (%).

^c^ P-values were calculated using the chi-squared test.

**Table 4.** Incidence of Hypotension and Severe Hypotension During Surgery by Group ^a,^ ^b^

Groups	Number	Hypotension	Severe Hypotension
**1**	955	175 (18.32)	19 (1.99)
**2**	329	69 (20.97)	15 (4.56)
**3**	2321	508 (21.89)	99 (4.27)
**4**	1368	412 (30.12)	86 (6.29)

^a^ Hypotension is defined as mean arterial pressure < 60 mmHg, and severe hypotension is defined as mean arterial pressure < 50 mmHg.

^b^ Values are expressed as the N. (%).

To further assess the incidences of hypotension and severe hypotension among the different groups, a logistic regression analysis was conducted (Table 5). The multivariable analysis revealed that group 4 had significantly higher incidences of both hypotension (OR = 1.407; 95% CI = 1.126 - 1.758; P = 0.003) and severe hypotension (OR = 1.940; 95% CI = 1.113 - 3.381; P = 0.019) compared with the reference group (group 1). Group 3 also demonstrated a higher incidence of hypotension than group 1.

**Table 5.** Logistic Regression Analysis Results for the Incidence of Hypotension and Severe Hypotension During Surgery ^a,^ ^b^

Variables	Univariable		Multivariable		
	OR	P-Value	OR	95% CI	P-Value
**Hypotension (n = 1164)**					
Male	Reference				
Female	0.984	0.813			
Age	1.025	< 0.001	1.025	1.020 - 1.031	< 0.001
Weight	0.989	< 0.001	0.995	0.988 - 1.001	0.105
Height	0.994	0.051	1.003	0.999 - 1.007	0.189
Group 1	Reference				
Group 2	1.125	0.457	0.949	0.686 - 1.313	0.752
Group 3	1.211	0.045	1.149	0.939 - 1.405	0.177
Group 4	1.831	< 0.001	1.407	1.126 - 1.758	0.003
**Severe hypotension (n = 219)**					
Male	Reference				
Female	0.989	0.938			
Age	1.041	< 0.001	1.042	1.028 - 1.056	< 0.001
Weight	0.986	0.018	1.000	0.985 - 1.015	0.982
Height	0.980	< 0.001	0.994	0.978 - 1.010	0.452
Group 1	Reference				
Group 2	2.388	0.013	1.652	0.805 - 3.393	0.171
Group 3	2.257	0.001	1.807	1.062 - 3.075	0.029
Group 4	3.396	< 0.001	1.940	1.113 - 3.381	0.019

Abbreviations: OR, odds ratio; CI, confidence interval.

^a^ Hypotension is defined as mean arterial pressure < 60 mmHg, and severe hypotension is defined as mean arterial pressure < 50 mmHg.

^b^ Cox and Snell R2 = 0.016, Nagelkerke R2 = 0.055, Hsomer and Lemshow P-value = 0.227, method = enter.

Table 6 shows the results of logistic regression analysis examining variables that contributed to the development of hypotension and severe hypotension during surgery. In terms of antihypertensive medication use, patients taking one antihypertensive drug and those taking two or more were compared, with patients not taking any medication serving as the reference. In the univariable analysis, each variable was assessed individually, while the multivariable analysis used a backward elimination method for statistically significant variables (P < 0.05) identified in the univariable analysis. In total, 1,164 patients (23.4%) developed hypotension during surgery. The risk of developing hypotension significantly increased with age (OR = 1.028; 95% CI = 1.021 - 1.034; P < 0.001) and the duration of anesthesia (OR = 1.003; 95% CI = 1.002 - 1.004; P < 0.001). The risk of intraoperative hypotension was lower during lumbar spine surgery compared to cervical spine surgery (OR 0.564, 95% CI 0.467 - 0.681, P < 0.001), and the occurrence of severe hypotension was also less significant in both lumbar and thoracic procedures than in cervical procedures (lumbar: OR 0.339, 95% CI 0.239 - 0.480, P < 0.001; thoracic: OR 0.538, 95% CI 0.322 - 0.900, P = 0.018). Taking a single antihypertensive medication did not increase the intraoperative hypotension risk, but the use of two or more drugs significantly did (OR = 1.0601; 95% CI = 1.274 - 2.011; P < 0.001). An additional analysis focused on severe hypotension indicated that the prone position significantly increased the likelihood of severe hypotension (OR = 1.505; 95% CI = 1.029 - 2.200; P = 0.035), which differed from the factors associated with general hypotension.

**Table 6.** Logistic Regression Analysis Results for Variables Causing Hypotension and Severe Hypotension During Surgery ^a,^ ^b^

Variables	Univariable		Multivariable	
	OR (95% CI)	P-Value	OR (95% CI)	P-Value
**Hypotension (n = 1164)**				
Duration of anesthesia	1.004 (1.003 - 1.004)	< 0.001	1.003 (1.002 - 1.004)	< 0.001
Amount of bleeding	1.000 (1.000 - 1.001)	< 0.001		
Age	1.032 (1.027 - 1.037)	< 0.001	1.028 (1.021 - 1.034)	< 0.001
Weight	0.993 (0.988 - 0.988)	0.012		
Height	0.991 (0.985 - 0.998)	0.013	1.014 (1.006 - 1.022)	0.001
Sex (F)	1.021 (0.895 - 1.165)	0.760		
DM	1.504 (1.278 - 1.770)	< 0.001	1.178 (0.991 - 1.401)	0.064
Hypertension	1.502 (1.312 - 1.719)	< 0.001	0.789 (0.641 - 0.971)	0.025
Cardiac disease	1.860 (1.492 - 2.318)	< 0.001		
Surgical position (prone)	1.416 (1.209 - 1.659)	< 0.001	1.158 (0.982 - 1.365)	0.082
Surgical region				
Cervical	Reference			
Lumbar	0.887 (0.764 - 1.031)	0.118	0.564 (0.467 - 0.681)	< 0.001
Thoracic	1.608 (1.249 - 2.069)	< 0.001	0.758 (0.566 - 1.016)	0.064
Antihypertensive drug count (0)	Reference			
Antihypertensive drug count (1)	1.390 (1.161 - 1.666)	< 0.001	1.163 (0.925 - 1.462)	0.196
Antihypertensive drug count (2 or more)	2.032 (1.741 - 2.372)	< 0.001	1.601 (1.274 - 2.011)	< 0.001
**Severe hypotension (n = 219)**				
Duration of anesthesia	1.004 (1.003 - 1.005)	< 0.001	1.003 (1.002 - 1.005)	< 0.001
Amount of bleeding	1.000 (1.000 - 1.000)	< 0.001		
Age	1.048 (1.036 - 1.060)	< 0.001	1.041 (1.027 - 1.055)	< 0.001
Weight	0.991 (0.980 - 1.003)	0.133		
Height	0.985 (0.972 - 0.999)	0.041	1.014 (0.998 - 1.030)	0.086
Sex (F)	1.033 (0.787 - 1.357)	0.815		
DM	2.007 (1.539 - 2.802)	< 0.001	1.572 (1.149 - 2.151)	0.005
Hypertension	1.694 (1.290 - 2.224)	< 0.001		
Cardiac disease	1.644 (1.077 - 2.511)	0.021		
Surgical position (prone)	1.941 (1.339 - 2.814)	< 0.001	1.505 (1.029 - 2.200)	0.035
Surgical region				
Cervical	Reference			
Lumbar	0.713 (0.527 - 0.964)	0.028	0.339 (0.239 - 0.480)	< 0.001
Thoracic	1.563 (0.991 - 2.465)	0.055	0.538 (0.322 - 0.900)	0.018
Antihypertensive drug count (0)	Reference			
Antihypertensive drug count (1)	1.234 (0.830 - 1.833)	0.299	0.753 (0.498 - 1.138)	0.178
Antihypertensive drug count (2 or more)	2.369 (1.758 - 3.192)	< 0.001	1.261 (0.908 - 1.751)	0.166

Abbreviations: OR, odds ratio; CI, confidence interval; DM, diabetes mellitus.

^a^ Hypotension is defined as a mean arterial pressure < 60 mmHg, and severe hypotension is defined as a mean arterial pressure < 50 mmHg.

^b^ Cox and Snell R2 = 0.025, Nagelkerke R2 = 0.081, Hosmer and Lemeshow P-value = 0.113.

## 5. Discussion

We investigated the incidence of intraoperative hypotension in patients undergoing spine surgery and examined whether factors such as surgical position, preoperative antihypertensive medication, and other patient characteristics affect it. The preoperative use of antihypertensive medications, particularly BBs, CCBs, ARBs, and diuretics, increased the likelihood of hypotension during surgery. Angiotensin-converting enzyme inhibitors were included in the analysis, but did not show a statistically significant association with intraoperative hypotension. Notably, the use of two or more antihypertensive medications significantly elevated the risk of intraoperative hypotension compared with no use of antihypertensive medications prior to surgery. Additionally, the prone position was found to be a potential risk factor for increased severe hypotension frequency. Our results also suggested that older patients and those with a higher ASA-PS grade experience a greater incidence of hypotension during surgery, corroborating the findings of a previous study (5). In our study, cervical spine surgery was associated with a higher incidence of intraoperative hypotension and severe hypotension compared to lumbar or thoracic spine surgery. Although direct comparisons between spinal regions are limited in the literature, a large-scale retrospective cohort study by Chiang et al. similarly reported a high incidence of intraoperative hypotension during cervical spine decompression procedures, with 43.4% of patients requiring vasopressor administration. The risk was particularly elevated in patients undergoing upper cervical spine (C_0_ - C_2_) surgery and those with a history of chronic hypertension. These findings may be attributable, at least in part, to impaired autonomic regulation associated with cervical spinal pathology or surgical manipulation (14).

In this study, a history of hypertension was not identified as a risk factor for intraoperative hypotension. Univariable analysis indicated that it was associated with an increased incidence of hypotension during surgery, but multivariable analysis yielded contrasting results. This discrepancy may be attributed to multicollinearity or confounding effects that arise when multiple independent variables are considered simultaneously. Consequently, the findings of the multivariable and univariable analyses were divergent.

However, it was found that taking antihypertensive medications before surgery may influence the incidence of intraoperative hypotension. Among patients who experienced hypotension during surgery, the use of BB, CCB, ARB, and diuretics was significantly higher than in those who did not develop hypotension. Most antihypertensive medications have the potential side-effect of inducing hypotension. According to previous studies, BBs should be maintained without interruption during the surgical period due to their beneficial effects, such as reducing the incidence of myocardial infarction in noncardiac surgeries; however, they increase the risk of bradycardia and hypotension during surgery (15, 16). Calcium channel blockers reduce myocardial contractility and elevate the risk of hypotension due to their vasodilatory effects (17). Diuretics can induce hypovolemia, which may be exacerbated by the administration of anesthetic agents (18). Angiotensin-converting enzyme inhibitors and ARBs lower blood pressure by inhibiting the renin-angiotensin-aldosterone system, which regulates vascular tone, systemic outflow, renal tubular and vascular function, and pressure natriuresis (19). These medications cause transient hypotension during surgery, leading to recommendations to discontinue them on the day of surgery, although this remains a topic of debate (4). Some studies indicate that continuing these medications on the day of surgery does not significantly impact major postoperative complications such as death, stroke, or myocardial infarction (20, 21). The most recent American Heart Association guidelines also suggest maintaining these medications on the day of surgery (16). In our hospital, however, it is recommended that patients discontinue ACE inhibitors on the day of surgery. In this study, when comparing the group that experienced hypotension during surgery with the group that did not, there was no significant difference in the number of patients taking ACE inhibitors. Although a precise analysis was challenging, we hypothesize that the discrepancy between our findings and those of existing studies may be attributable to our hospital's policy.

An intriguing result of our study was that the incidence of intraoperative hypotension was significantly higher among patients who were taking two or more antihypertensive medications prior to surgery. When comparing the groups — those who did not take antihypertensive medication, those who took only one medication, and those who took two or more medications — no statistical difference in the frequency of intraoperative hypotension between the group that took only one medication and the group that did not take any was observed. Conversely, patients who were taking two or more antihypertensive medications exhibited a statistically significantly higher incidence of intraoperative hypotension. Hypertension is a prevalent chronic condition, and to enhance treatment efficacy, dual or combination therapy with antihypertensive drugs is increasingly utilized (22). Combination therapy offers advantages such as more effective blood pressure reduction and a decrease in side effects, which can result from decreased dosages of individual drugs. However, side effects, including hypotension, have also been reported (23, 24). In this study, the number of patients receiving combination therapy was 1,095, which was higher than the number of patients receiving monotherapy (n = 830). Although numerous studies have examined the effects of individual antihypertensive medications on perioperative outcomes or the occurrence of hypotension during surgery, the impact of antihypertensive combination therapy on hemodynamic changes during the perioperative period remains unclear (4). The findings of this study revealed a greater incidence of hypotension during surgery among patients taking two or more antihypertensive medications. We hypothesize that this may be due to the distinct mechanisms by which individual antihypertensive drugs lower blood pressure, which may synergistically enhance hypotensive effects during general anesthesia. Further research is warranted to investigate the effects of combination therapy with antihypertensive drugs on hemodynamic variables during the perioperative period and to elucidate the underlying mechanisms contributing to these effects.

In this study, the incidence of hypotension in patients positioned prone was 24.91%, which was significantly higher than the 19.06% incidence observed in the supine position. The difference in the incidence of severe hypotension was even more pronounced, with rates of 2.65% in the supine position compared with 5.01% in the prone position. Multivariable analysis using logistic regression confirmed these results, indicating that the prone position is a significant risk factor for the development of severe hypotension. Previous studies have also documented the occurrence of hypotension in prone patients, attributing it to decreased venous return resulting from vena cava compression, as well as reductions in stroke volume and Cardiac Index (25, 26). Additionally, the hypotensive effects of general anesthetic agents may exacerbate the severity of hypotension (27, 28). We further analyzed risk factors for severe hypotension based on the degree of hypotension, which is anticipated to aid in anesthetic management during spinal surgery.

This study has some limitations. First, although bleeding volume was included in the analysis and was significantly associated with hypotension, it was not a primary variable of interest. Given its close relationship with blood pressure, it may have acted as a confounding factor. However, the main findings of this study — namely the association between surgical position, preoperative antihypertensive medication use, and intraoperative hypotension — remained statistically significant, regardless of bleeding volume, suggesting the robustness of our results. Second, patients on multiple antihypertensives were grouped only by the number of drugs (0, 1, 2 or more), rather than analyzing specific combinations. Comprehensive analysis of all combinations of antihypertensive drugs was not feasible due to the extensive number of two or more combination therapies, making individual analysis impractical. Future studies should directly compare specific drug classes and investigate potential synergistic or antagonistic interactions in combination therapies. Third, as this study was retrospective in nature, there was limited control over various surgical conditions, including anesthesia methods and surgical procedures. Notably, the doses of remifentanil and propofol are known to influence blood pressure however, this retrospective design limited our ability to quantify remifentanil and propofol dosages, which are known to influence hemodynamics and may act as unmeasured confounders (6, 27). According to our hospital’s policy, remifentanil is administered at a low dose of 0.03 - 0.1 mcg/kg/min to maintain anesthesia. If blood pressure drops, the dose of remifentanil is first reduced to maintain appropriate blood pressure. Therefore, it is difficult to determine if the amount of remifentanil used was the main cause of the difference in hypotension incidence. To address this limitation, we employed an optimal logistic regression model utilizing a backward elimination method. In this approach, all independent variables that could potentially affect blood pressure were included in the regression analysis. Subsequently, variables with the least impact were systematically removed until only statistically significant variables remained, ensuring that excluded variables were not reselected.

### 5.1. Conclusions

In summary, we examined the effects of preoperative antihypertensive medication use and surgical positioning on the incidence of intraoperative hypotension in patients undergoing spine surgery. The use of two or more antihypertensive medications prior to surgery may elevate the risk of intraoperative hypotension. Additionally, performing surgery in the prone position may contribute to an increased likelihood of severe hypotension. Therefore, caution is warranted during general anesthesia in these patients. These findings suggest that patients undergoing spine surgery in the prone position while receiving two or more antihypertensive agents may benefit from enhanced perioperative monitoring and hemodynamic optimization strategies.

## Data Availability

The dataset presented in the study is available on request from the corresponding author during submission or after publication. The data are not publicly available due to privacy protection and ethical restrictions.

## Clinical Trial Registration Code:

Clinical Research Information Service (CRIS) number: KCT0009892.
